# Supplementary material for: Environmental impacts, water footprint and cumulative energy demand of match industry in Pakistan
Source: PLoS One. 2021 May 20;16(5):e0251928. doi: 10.1371/journal.pone.0251928 (PMC8136843; doi:10.1371/journal.pone.0251928)
Supplement: S1 Appendix — (DOCX) [file pone.0251928.s001.docx]

**S1 Appendix**

**S1 Table.** Emission inventory to water for one carton of match produced by match industries in KP during 2019-20.

| Substance | Compartment | Unit | Total |
| --- | --- | --- | --- |
| 1-Butanol | Water | mg | 2.747 |
| 1-Propanol | Water | µg | 2.907 |
| Acetic acid | Water | mg | 27.765 |
| Acetone | Water | µg | 26.189 |
| Aluminium | Water | g | 9.563 |
| Arsenic | Water | mg | 48.655 |
| Benzene | Water | mg | 56.894 |
| BOD5, Biological Oxygen Demand | Water | g | 164.118 |
| Calcium | Water | g | 146.214 |
| Carbon | Water | µg | 24.020 |
| Chloramine | Water | µg | 21.340 |
| Chlorate | Water | g | 1.107 |
| Chlorine | Water | mg | 14.324 |
| Chloroform | Water | µg | 1.078 |
| Chromium | Water | mg | 28.307 |
| Cobalt | Water | mg | 144.551 |
| COD, Chemical Oxygen Demand | Water | g | 180.081 |
| Copper | Water | g | 1.800 |
| Cyanide | Water | mg | 27.569 |
| DOC, Dissolved Organic Carbon | Water | g | 59.743 |
| Fluoride | Water | g | 1.744 |
| Formic acid | Water | ng | 471.262 |
| Hydrogen chloride | Water | mg | 990.573 |
| Hydroxide | Water | mg | 1.702 |
| Iodide | Water | mg | 63.239 |
| Lead | Water | mg | 159.640 |
| Lithium | Water | mg | 514.944 |
| Nitrogen | Water | mg | 388.783 |
| Organic carbon | Water | µg | 56.808 |
| Paraffins | Water | µg | 112.729 |
| Phosphorus | Water | mg | 139.236 |
| Potassium | Water | g | 36.994 |
| Silver | Water | mg | 2.786 |
| Sodium | Water | g | 342.667 |
| Sulfur | Water | mg | 442.391 |
|  |  |  |  |
|  |  |  |  |

**S2 Table.** Emission inventory to soil for one carton of match produced by match industries in KP during 2019-20.

| Substance | Compartment | Unit | Total |
| --- | --- | --- | --- |
| Acetamide | Soil | µg | 9.574 |
| Aluminium | Soil | mg | 354.466 |
| Antimony | Soil | µg | 41.400 |
| Arsenic | Soil | µg | 236.611 |
| Cadmium | Soil | µg | 136.348 |
| Calcium | Soil | g | 1.525 |
| Carbon | Soil | g | 1.153 |
| Chlorine | Soil | mg | 10.767 |
| Chromium | Soil | mg | 2.381 |
| Cobalt | Soil | µg | 289.361 |
| Copper | Soil | mg | 3.635 |
| Fluoride | Soil | mg | 16.471 |
| Iron | Soil | g | 1.036 |
| Lead | Soil | mg | 4.005 |
| Lithium | Soil | µg | 26.982 |
| Manganese | Soil | mg | 31.283 |
| Nickel | Soil | µg | 469.052 |
| Nitrate | Soil | mg | 31.094 |
| Nitrogen | Soil | µg | 395.788 |
| Oils, biogenic | Soil | g | 1.585 |
| Organic carbon | Soil | µg | 56.808 |
| Phosphorus | Soil | mg | 24.317 |
| Potassium | Soil | mg | 162.620 |
| Silicon | Soil | mg | 140.146 |
| Silver | Soil | µg | 2.074 |
| Strontium | Soil | mg | 3.497 |
| Sodium | Soil | mg | 962.382 |
| Selenium | Soil | µg | 413.727 |
| Sulfur | Soil | mg | 209.192 |
| Tin | Soil | ng | 362.772 |
| Titanium | Soil | mg | 9.023 |
| Vanadium | Soil | µg | 54.655 |
| Zinc | Soil | mg | 158.974 |

**S3 Table.** Emission inventory to air for one carton of match produced by match industries in KP during 2019-20.

| Substance | Compartment | Unit | Total |
| --- | --- | --- | --- |
| 1-Butanol | Air | µg | 1.584 |
| 1-Propanol | Air | µg | 526.755 |
| Acetamide | Air | µg | 4.658 |
| Acetic acid | Air | Mg | 224.355 |
| Aluminium | Air | G | 1.907 |
| Ammonia | Air | G | 1.258 |
| Benzene | Air | Mg | 298.244 |
| Cadmium | Air | Mg | 1.785 |
| Calcium | Air | Mg | 82.871 |
| Carbon | Air | µg | 7.019 |
| Carbon dioxide | Air | Mg | 571.461 |
| Chlorine | Air | Mg | 143.784 |
| Chloroform | Air | µg | 109.627 |
| Chromium | Air | Mg | 21.647 |
| Cobalt | Air | Mg | 1.355 |
| Copper | Air | Mg | 199.484 |
| Cyanide | Air | Mg | 29.958 |
| Formic acid | Air | Mg | 9.100 |
| Helium | Air | Mg | 27.312 |
| Hydrogen | Air | G | 2.183 |
| Hydrogen chloride | Air | G | 1.533 |
| Iodine | Air | Mg | 4.962 |
| Iron | Air | Mg | 845.797 |
| Manganese | Air | Mg | 13.495 |
| Methane | Air | µg | 24.187 |
| Nickel | Air | Mg | 18.327 |
| Noble gases, radioactive, unspecified | Air | kBq | 143.183 |
| Nitrate | Air | Mg | 16.059 |
| Organic carbon | Air | µg | 17.458 |
| Ozone | Air | Mg | 37.476 |
| Paraffins | Air | µg | 38.844 |
| Phosphorus | Air | Mg | 2.101 |
| Potassium | Air | Mg | 116.717 |
| Sodium | Air | Mg | 59.613 |
| Xylene | Air | Mg | 119.769 |
| Zinc | Air | Mg | 131.838 |

**S4 Table.** Specification per substance to global warming potential.

| Substance | Compartment | Unit | Total |
| --- | --- | --- | --- |
| Total of all compartments |  | kg CO2 eq | 43.69248195 |
| Carbon dioxide | Air | kg CO2 eq | 0.000571461 |
| Carbon dioxide, fossil | Air | kg CO2 eq | 41.46060491 |
| Carbon dioxide, land transformation | Air | kg CO2 eq | 0.17469465 |
| Carbon monoxide, fossil | Air | kg CO2 eq | 0.173811473 |
| Chloroform | Air | kg CO2 eq | 3.28882E-06 |
| Dinitrogen monoxide | Air | kg CO2 eq | 0.553446712 |
| Ethane, 1,1-difluoro-, HFC-152a | Air | kg CO2 eq | 6.25041E-05 |
| Ethane, hexafluoro-, HFC-116 | Air | kg CO2 eq | 0.001455365 |
| Methane | Air | kg CO2 eq | 5.563E-07 |
| Methane, biogenic | Air | kg CO2 eq | 0.008720928 |
| Methane, bromo-, Halon 1001 | Air | kg CO2 eq | 4.67992E-13 |
| Methane, chlorodifluoro-, HCFC-22 | Air | kg CO2 eq | 0.001331626 |
| Methane, dichloro-, HCC-30 | Air | kg CO2 eq | 9.42686E-07 |
| Methane, dichlorodifluoro-, CFC-12 | Air | kg CO2 eq | 0.000484594 |
| Methane, dichlorofluoro-, HCFC-21 | Air | kg CO2 eq | 9.57431E-09 |
| Methane, fossil | Air | kg CO2 eq | 1.276281504 |
| Methane, monochloro-, R-40 | Air | kg CO2 eq | 2.02073E-06 |
| Methane, tetrachloro-, CFC-10 | Air | kg CO2 eq | 0.000174707 |
| Methane, tetrafluoro-, CFC-14 | Air | kg CO2 eq | 0.009473614 |
| Methane, trichlorofluoro-, CFC-11 | Air | kg CO2 eq | 3.35137E-07 |
| Methane, trifluoro-, HFC-23 | Air | kg CO2 eq | 0.000174078 |
| Sulfur hexafluoride | Air | kg CO2 eq | 0.025299084 |

**S5 Table.** Specification per substance to abiotic depletion.

| Substance | Compartment | Unit | Total |
| --- | --- | --- | --- |
| Total of all compartments |  | kg Sb eq | 0.330677795 |
| Aluminium | Raw | kg Sb eq | 2.61051E-10 |
| Argon | Raw | kg Sb eq | 1.47285E-09 |
| Borax | Raw | kg Sb eq | 4.41467E-09 |
| Bromine | Raw | kg Sb eq | 5.81031E-08 |
| Cadmium | Raw | kg Sb eq | 9.82531E-05 |
| Calcite | Raw | kg Sb eq | 3.21023E-10 |
| Chromium | Raw | kg Sb eq | 5.53123E-06 |
| Coal, hard | Raw | kg Sb eq | 0.035902966 |
| Cobalt | Raw | kg Sb eq | 1.69552E-06 |
| Dolomite | Raw | kg Sb eq | 2.47863E-12 |
| Fluorine | Raw | kg Sb eq | 5.27291E-07 |
| Gas, natural/m3 | Raw | kg Sb eq | 0.031313875 |
| Gold | Raw | kg Sb eq | 1.20889E-05 |
| Gypsum | Raw | kg Sb eq | 2.87565E-07 |
| Iodine | Raw | kg Sb eq | 7.93993E-08 |
| Iron | Raw | kg Sb eq | 7.12297E-08 |
| Lead | Raw | kg Sb eq | 6.69986E-05 |
| Lithium | Raw | kg Sb eq | 5.26451E-11 |
| Manganese | Raw | kg Sb eq | 4.11E-08 |
| Oil, crude | Raw | kg Sb eq | 0.255590517 |
| Sodium chloride | Raw | kg Sb eq | 1.07716E-08 |
| Sodium nitrate | Raw | kg Sb eq | 1.40725E-21 |
| Sulfur | Raw | kg Sb eq | 4.21498E-07 |
| Tin | Raw | kg Sb eq | 1.16954E-06 |
| Uranium | Raw | kg Sb eq | 1.00726E-07 |
| Xenon | Raw | kg Sb eq | 0.000176787 |
| Zinc | Raw | kg Sb eq | 8.8627E-06 |

**S6 Table.** Specification per substance to acidification.

| Substance | Compartment | Unit | Total |
| --- | --- | --- | --- |
| Total of all compartments |  | kg SO2 eq | 0.142210803 |
| Ammonia | Air | kg SO2 eq | 0.002013262 |
| Nitrogen oxides | Air | kg SO2 eq | 0.043228879 |
| Sulfur dioxide | Air | kg SO2 eq | 0.096968661 |

**S7 Table.** Specification per substance to eutrophication**.**

| Substance | Compartment | Unit | Total |
| --- | --- | --- | --- |
| Total of all compartments |  | kg PO4--- eq | 0.033766139 |
| Ammonia | Air | kg PO4--- eq | 0.000440401 |
| Ammonium carbonate | Air | kg PO4--- eq | 6.80352E-10 |
| Ammonium, ion | Water | kg PO4--- eq | 5.14231E-05 |
| COD, Chemical Oxygen Demand | Water | kg PO4--- eq | 0.003961778 |
| Nitrate | Air | kg PO4--- eq | 1.60589E-06 |
| Nitrate | Water | kg PO4--- eq | 0.000947905 |
| Nitrate | Soil | kg PO4--- eq | 3.10937E-06 |
| Nitrite | Water | kg PO4--- eq | 1.1998E-07 |
| Nitrogen | Water | kg PO4--- eq | 0.000163289 |
| Nitrogen | Soil | kg PO4--- eq | 1.66231E-07 |
| Nitrogen oxides | Air | kg PO4--- eq | 0.011239509 |
| Phosphate | Water | kg PO4--- eq | 0.016449931 |
| Phosphoric acid | Air | kg PO4--- eq | 2.19422E-11 |
| Phosphorus | Air | kg PO4--- eq | 6.4292E-06 |
| Phosphorus | Water | kg PO4--- eq | 0.000426061 |
| Phosphorus | Soil | kg PO4--- eq | 7.44095E-05 |

**S8 Table.** Specification per substance to freshwater aquatic ecotoxicity.

| Substance | | Compartment | | Unit | Total |
| --- | --- | --- | --- | --- | --- |
| Total of all compartments | |  | | kg 1,4-DB eq | 7.194323308 |
| Antimony | | Air | | kg 1,4-DB eq | 0.001343116 |
| Antimony | | Water | | kg 1,4-DB eq | 0.000587859 |
| Antimony | | Soil | | kg 1,4-DB eq | 4.13169E-07 |
| Arsenic | | Air | | kg 1,4-DB eq | 0.000216123 |
| Arsenic | | Water | | kg 1,4-DB eq | 0.010049563 |
| Arsenic | | Soil | | kg 1,4-DB eq | 3.17059E-05 |
| Barium | | Air | | kg 1,4-DB eq | 0.006122404 |
| Barium | | Water | | kg 1,4-DB eq | 0.174072713 |
| Barium | | Soil | | kg 1,4-DB eq | 0.018374227 |
| Benzene | | Air | | kg 1,4-DB eq | 2.4963E-08 |
| Benzene | | Water | | kg 1,4-DB eq | 4.65198E-06 |
| Chloroform | | Air | | kg 1,4-DB eq | 1.04365E-11 |
| Chloroform | | Water | | kg 1,4-DB eq | 4.56088E-11 |
| Cobalt | | Air | | kg 1,4-DB eq | 0.000865974 |
| Cobalt | | Water | | kg 1,4-DB eq | 0.492919844 |
| Cobalt | | Soil | | kg 1,4-DB eq | 0.000494807 |
| Copper | | Air | | kg 1,4-DB eq | 0.044285453 |
| Copper | | Water | | kg 1,4-DB eq | 2.087905508 |
| Copper | | Soil | | kg 1,4-DB eq | 0.002162847 |
| Ethene | | Air | | kg 1,4-DB eq | 1.78769E-15 |
| Ethene | | Water | | kg 1,4-DB eq | 1.94656E-06 |
| Lead | | Air | | kg 1,4-DB eq | 0.000100082 |
| Lead | | Water | | kg 1,4-DB eq | 0.001518628 |
| Lead | | Soil | | kg 1,4-DB eq | 2.61505E-05 |
| Mercury | | Air | | kg 1,4-DB eq | 0.000370546 |
| Mercury | | Water | | kg 1,4-DB eq | 0.002264027 |
| Mercury | | Soil | | kg 1,4-DB eq | 1.62922E-06 |
| Nickel | | Air | | kg 1,4-DB eq | 0.011527387 |
| Nickel | | Water | | kg 1,4-DB eq | 1.967218034 |
| Nickel | | Soil | | kg 1,4-DB eq | 0.000792699 |
| Tin | | Air | | kg 1,4-DB eq | 6.55934E-05 |
| Tin | | Water | | kg 1,4-DB eq | 0.000830837 |
| Tin | | Soil | | kg 1,4-DB eq | 2.50313E-09 |
| Vanadium | | Air | | kg 1,4-DB eq | 0.031341253 |
| Vanadium | | Water | | kg 1,4-DB eq | 0.768730555 |
| Vanadium | | Soil | | kg 1,4-DB eq | 0.000254145 |
| Zinc | | Air | | kg 1,4-DB eq | 0.002346721 |
|  |  | |  |  |  |
|  |  | |  |  |  |

**S9 Table.** Specification per substance to human health.

| Substance | Compartment | Unit | Total |
| --- | --- | --- | --- |
| Total of all compartments |  | kg 1,4-DB eq | 17.84399316 |
| Ammonia | Air | kg 1,4-DB eq | 0.000125829 |
| Arsenic | Air | kg 1,4-DB eq | 1.519411963 |
| Arsenic | Water | kg 1,4-DB eq | 0.046425781 |
| Arsenic | Soil | kg 1,4-DB eq | 0.001231544 |
| Benzene | Air | kg 1,4-DB eq | 0.56666372 |
| Benzene | Water | kg 1,4-DB eq | 0.094399783 |
| Chloroform | Air | kg 1,4-DB eq | 1.39227E-06 |
| Chloroform | Water | kg 1,4-DB eq | 1.34778E-08 |
| Chromium | Soil | kg 1,4-DB eq | 0.002237519 |
| Cobalt | Air | kg 1,4-DB eq | 0.023716038 |
| Cobalt | Water | kg 1,4-DB eq | 0.013978113 |
| Cobalt | Soil | kg 1,4-DB eq | 5.62642E-05 |
| Copper | Air | kg 1,4-DB eq | 0.857781303 |
| Copper | Water | kg 1,4-DB eq | 0.002414082 |
| Copper | Soil | kg 1,4-DB eq | -8.28494E-05 |
| Ethene | Air | kg 1,4-DB eq | 7.96334E-05 |
| Ethene | Water | kg 1,4-DB eq | 5.65799E-05 |
| Lead | Air | kg 1,4-DB eq | 0.019474284 |
| Lead | Water | kg 1,4-DB eq | 0.002081826 |
| Lead | Soil | kg 1,4-DB eq | 0.001686198 |
| Mercury | Air | kg 1,4-DB eq | 0.007025178 |
| Mercury | Water | kg 1,4-DB eq | 0.001908353 |
| Mercury | Soil | kg 1,4-DB eq | 7.56132E-06 |
| Nickel | Air | kg 1,4-DB eq | 0.641428552 |
| Nickel | Water | kg 1,4-DB eq | 0.201076484 |
| Nickel | Soil | kg 1,4-DB eq | -0.000432282 |
| Tin | Air | kg 1,4-DB eq | 4.46758E-05 |
| Tin | Water | kg 1,4-DB eq | 1.40917E-06 |
| Tin | Soil | kg 1,4-DB eq | 4.75232E-09 |
| Zinc | Air | kg 1,4-DB eq | 0.013711182 |
| Zinc | Water | kg 1,4-DB eq | 0.001864206 |
| Zinc | Soil | kg 1,4-DB eq | 1.97725E-05 |

**S10 Table.** Specification per substance to marine toxicity.

| Substance | Compartment | Unit | Total |
| --- | --- | --- | --- |
| Total of all compartments |  | kg 1,4-DB eq | 11758.79786 |
| Antimony | Air | kg 1,4-DB eq | 11.95084318 |
| Antimony | Water | kg 1,4-DB eq | 0.808678681 |
| Antimony | Soil | kg 1,4-DB eq | 0.000567176 |
| Arsenic | Air | kg 1,4-DB eq | 1.008575183 |
| Arsenic | Water | kg 1,4-DB eq | 5.813878801 |
| Arsenic | Soil | kg 1,4-DB eq | 0.018242733 |
| Benzene | Air | kg 1,4-DB eq | 8.35083E-07 |
| Benzene | Water | kg 1,4-DB eq | 2.26545E-07 |
| Cadmium | Air | kg 1,4-DB eq | 1.980999313 |
| Cadmium | Water | kg 1,4-DB eq | 4.762763194 |
| Cadmium | Soil | kg 1,4-DB eq | 0.015271015 |
| Chloroform | Air | kg 1,4-DB eq | 6.48994E-09 |
| Chloroform | Water | kg 1,4-DB eq | 6.27525E-11 |
| Cobalt | Air | kg 1,4-DB eq | 7.372299787 |
| Cobalt | Water | kg 1,4-DB eq | 633.1352516 |
| Cobalt | Soil | kg 1,4-DB eq | 0.63659386 |
| Copper | Air | kg 1,4-DB eq | 178.1392335 |
| Copper | Water | kg 1,4-DB eq | 419.9297469 |
| Copper | Soil | kg 1,4-DB eq | 0.43620451 |
| Ethene | Air | kg 1,4-DB eq | 9.91354E-15 |
| Ethene | Water | kg 1,4-DB eq | 2.40508E-09 |
| Lead | Air | kg 1,4-DB eq | 0.293990792 |
| Lead | Water | kg 1,4-DB eq | 0.195320882 |
| Lead | Soil | kg 1,4-DB eq | 0.003015521 |
| Nickel | Air | kg 1,4-DB eq | 68.907753 |
| Nickel | Water | kg 1,4-DB eq | 1366.930961 |
| Nickel | Soil | kg 1,4-DB eq | 0.548791276 |
| Tin | Air | kg 1,4-DB eq | 0.193939657 |
| Tin | Water | kg 1,4-DB eq | 0.100189222 |
| Tin | Soil | kg 1,4-DB eq | 3.02552E-07 |
| Zinc | Air | kg 1,4-DB eq | 8.872716509 |
| Zinc | Water | kg 1,4-DB eq | 45.93645726 |
| Zinc | Soil | kg 1,4-DB eq | 1.14620197 |

**S11 Table.** Specification per substance to Ozone layer depletion (OLD).

| Substance | Compartment | Unit | Total |
| --- | --- | --- | --- |
| Total of all compartments |  | kg CFC-11 eq | 7.55358E-06 |
| Ethane, 1,1,1-trichloro-, HCFC-140 | Air | kg CFC-11 eq | 5.24552E-10 |
| Ethane, 1,1,2-trichloro-1,2,2-trifluoro-, CFC-113 | Air | kg CFC-11 eq | 2.18596E-08 |
| Ethane, 2-chloro-1,1,1,2-tetrafluoro-, HCFC-124 | Air | kg CFC-11 eq | 5.46573E-10 |
| Methane, bromo-, Halon 1001 | Air | kg CFC-11 eq | 3.46314E-14 |
| Methane, bromochlorodifluoro-, Halon 1211 | Air | kg CFC-11 eq | 7.54609E-08 |
| Methane, bromotrifluoro-, Halon 1301 | Air | kg CFC-11 eq | 7.16107E-06 |
| Methane, chlorodifluoro-, HCFC-22 | Air | kg CFC-11 eq | 2.66325E-08 |
| Methane, dichlorodifluoro-, CFC-12 | Air | kg CFC-11 eq | 3.74875E-08 |
| Methane, monochloro-, R-40 | Air | kg CFC-11 eq | 2.52591E-09 |
| Methane, tetrachloro-, CFC-10 | Air | kg CFC-11 eq | 1.16472E-07 |
| Methane, trichlorofluoro-, CFC-11 | Air | kg CFC-11 eq | 7.28559E-11 |

**S12 Table.** Specification per substance to photochemical oxidation**.**

| Substance | Compartment | Unit | Total |
| --- | --- | --- | --- |
| Total of all compartments |  | kg C2H4 eq | 0.010449254 |
| 1-Butanol | Air | kg C2H4 eq | 9.82014E-10 |
| 1-Pentene | Air | kg C2H4 eq | 3.67169E-10 |
| 1-Propanol | Air | kg C2H4 eq | 2.95509E-07 |
| 2-Propanol | Air | kg C2H4 eq | 6.90532E-07 |
| Acetic acid | Air | kg C2H4 eq | 2.17624E-05 |
| Acetone | Air | kg C2H4 eq | 2.13728E-06 |
| Benzene | Air | kg C2H4 eq | 6.50172E-05 |
| Butane | Air | kg C2H4 eq | 0.000259357 |
| Carbon monoxide, biogenic | Air | kg C2H4 eq | 2.91864E-05 |
| Carbon monoxide, fossil | Air | kg C2H4 eq | 0.002989115 |
| Chloroform | Air | kg C2H4 eq | 2.52143E-09 |
| Diethyl ether | Air | kg C2H4 eq | 1.66516E-12 |
| Ethane | Air | kg C2H4 eq | 5.06514E-05 |
| Ethanol | Air | kg C2H4 eq | 2.3182E-06 |
| Ethene | Air | kg C2H4 eq | 0.000125013 |
| Ethyl acetate | Air | kg C2H4 eq | 3.6038E-06 |
| Ethyne | Air | kg C2H4 eq | 2.95822E-06 |
| Formaldehyde | Air | kg C2H4 eq | 0.000595376 |
| Formic acid | Air | kg C2H4 eq | 2.912E-07 |
| Heptane | Air | kg C2H4 eq | 8.14845E-05 |
| Hexane | Air | kg C2H4 eq | 0.000259953 |
| Methane | Air | kg C2H4 eq | 1.45122E-10 |
| Methane, biogenic | Air | kg C2H4 eq | 2.61628E-06 |
| Methane, fossil | Air | kg C2H4 eq | 0.000332943 |
| Methanol | Air | kg C2H4 eq | 1.562E-05 |
| Methyl acetate | Air | kg C2H4 eq | 1.12861E-11 |
| Methyl formate | Air | kg C2H4 eq | 7.84943E-13 |
| Propane | Air | kg C2H4 eq | 0.00013497 |
| Sulfur dioxide | Air | kg C2H4 eq | 0.003878746 |

**S13 Table.** Specification per substance to terrestrial toxicity.

| Substance | Compartment | Unit | Total |
| --- | --- | --- | --- |
| Antimony | Air | kg 1,4-DB eq | 0.000220603 |
| Antimony | Water | kg 1,4-DB eq | 4.95353E-25 |
| Antimony | Soil | kg 1,4-DB eq | 5.17497E-08 |
| Arsenic | Air | kg 1,4-DB eq | 0.007029463 |
| Arsenic | Water | kg 1,4-DB eq | 5.08064E-22 |
| Arsenic | Soil | kg 1,4-DB eq | 0.000790282 |
| Barium | Air | kg 1,4-DB eq | 0.000695208 |
| Barium | Water | kg 1,4-DB eq | 4.07865E-22 |
| Barium | Soil | kg 1,4-DB eq | 0.001592966 |
| Benzene | Air | kg 1,4-DB eq | 4.65261E-09 |
| Benzene | Water | kg 1,4-DB eq | 7.07536E-10 |
| Chloroform | Air | kg 1,4-DB eq | 4.40702E-12 |
| Chloroform | Water | kg 1,4-DB eq | 4.22663E-14 |
| Cobalt | Air | kg 1,4-DB eq | 0.000147717 |
| Cobalt | Water | kg 1,4-DB eq | 3.88843E-22 |
| Cobalt | Soil | kg 1,4-DB eq | 6.45275E-05 |
| Copper | Air | kg 1,4-DB eq | 0.001394393 |
| Copper | Water | kg 1,4-DB eq | 7.31686E-24 |
| Copper | Soil | kg 1,4-DB eq | 5.23445E-05 |
| Ethene | Air | kg 1,4-DB eq | 1.68768E-16 |
| Ethene | Water | kg 1,4-DB eq | 9.68952E-17 |
| Lead | Air | kg 1,4-DB eq | 0.000654703 |
| Lead | Water | kg 1,4-DB eq | 8.34267E-26 |
| Lead | Soil | kg 1,4-DB eq | 0.000130152 |
| Mercury | Air | kg 1,4-DB eq | 0.033080289 |
| Mercury | Water | kg 1,4-DB eq | 0.001248432 |
| Mercury | Soil | kg 1,4-DB eq | 0.00010759 |
| Nickel | Air | kg 1,4-DB eq | 0.002125877 |
| Nickel | Water | kg 1,4-DB eq | 6.25746E-22 |
| Nickel | Soil | kg 1,4-DB eq | 0.000112104 |
| Tin | Air | kg 1,4-DB eq | 0.000371868 |
| Tin | Water | kg 1,4-DB eq | 6.40234E-26 |
| Tin | Soil | kg 1,4-DB eq | 1.08106E-08 |
| Zinc | Air | kg 1,4-DB eq | 0.001582059 |
| Zinc | Water | kg 1,4-DB eq | 8.36815E-24 |
| Zinc | Soil | kg 1,4-DB eq | 0.003910758 |
