# Supplementary material for: Environmental impacts, water footprint and cumulative energy demand of match industry in Pakistan
Source: PLoS One. 2021 May 20;16(5):e0251928. doi: 10.1371/journal.pone.0251928 (PMC8136843; doi:10.1371/journal.pone.0251928)
Supplement: Attachment — Submitted filename: Response to Reviewers.docx [file pone.0251928.s002.docx]

**Response to Reviewers**

PONE-D-21-08698
Environmental impacts, water footprint and cumulative energy demand of match industry in
Please include the following items when submitting your revised manuscript:

- A rebuttal letter that responds to each point raised by the academic editor and reviewer(s). You should upload this letter as a separate file labeled 'Response to Reviewers'.
- A marked-up copy of your manuscript that highlights changes made to the original version. You should upload this as a separate file labeled 'Revised Manuscript with Track Changes'.
- An unmarked version of your revised paper without tracked changes. You should upload this as a separate file labeled 'Manuscript'.

We look forward to receiving your revised manuscript.

Kind regards,

Bing Xue, Ph.D.
Academic Editor
PLOS ONE

Journal Requirements:

When submitting your revision, we need you to address these additional requirements.

1. Please ensure that your manuscript meets PLOS ONE's style requirements, including those for file naming. The PLOS ONE style templates can be found at https://journals.plos.org/plosone/s/file?id=wjVg/PLOSOne_formatting_sample_main_body.pdf and [https://journals.plos.org/plosone/s/file?id=ba62/PLOSOne_formatting_sample_title_authors_affiliations.pdf](about:blank)

**Answer: Revised manuscript is formatted according to PloS One style now.**

2. We suggest you thoroughly copyedit your manuscript for language usage, spelling, and grammar.

**Answer: The revised manuscript is thoroughly copyedited for language usage, spelling and grammar by one of my English native friend Dr. Alessandra Serafino.**  

3. Please include captions for your Supporting Information files at the end of your manuscript, and update any in-text citations to match accordingly

**Answer: Captions are provided for supporting information files at the end of the revised manuscript and all the citation and references are update and matched.**

Please review your reference list to ensure that it is complete and correct. If you have cited papers that have been retracted, please include the rationale for doing so in the manuscript text, or remove these references and replace them with relevant current references. Any changes to the reference list should be mentioned in the rebuttal letter that accompanies your revised manuscript. If you need to cite a retracted article, indicate the article’s retracted status in the References list and also include a citation and full reference for the retraction notice.

Answer: all the citations and references are double checked and corrected in the revised manuscript as per PLOS One author guidelines.

1) the introduction should be improved by adding more new literature, and please also try to outline your research question and potential contributions.

**Answer: the introduction part of the manuscript is revised and improved.**

2) please improve the discussion section

**Answer: Discussion section is improved in the revised manuscript.**

3) the language should be improved.

**Answer: Native English Friend improves the English language of the manuscript voluntarily.**

1. Is the manuscript technically sound, and do the data support the conclusions?

The manuscript must describe a technically sound piece of scientific research with data that supports the conclusions. Experiments must have been conducted rigorously, with appropriate controls, replication, and sample sizes. The conclusions must be drawn appropriately based on the data presented.

**Reviewer #1: Yes**

2. Has the statistical analysis been performed appropriately and rigorously?

**Reviewer #1: Yes**

3. Have the authors made all data underlying the findings in their manuscript fully available?

The [PLOS Data policy](http://www.plosone.org/static/policies.action#sharing) requires authors to make all data underlying the findings described in their manuscript fully available without restriction, with rare exception (please refer to the Data Availability Statement in the manuscript PDF file). The data should be provided as part of the manuscript or its supporting information, or deposited to a public repository. For example, in addition to summary statistics, the data points behind means, medians and variance measures should be available. If there are restrictions on publicly sharing data—e.g. participant privacy or use of data from a third party—those must be specified.

**Reviewer #1: No**

**All the data underlying the findings are attached as supplemental materials in the revised manuscript.**

4. Is the manuscript presented in an intelligible fashion and written in standard English?

PLOS ONE does not copyedit accepted manuscripts, so the language in submitted articles must be clear, correct, and unambiguous. Any typographical or grammatical errors should be corrected at revision, so please note any specific errors here.

**Reviewer #1: Yes**

4. Review Comments to the Author

   Reviewer #1: The article is accepted but before the publication of this article the author needs to make the required changes as explained above in the comments. The study contains an important factor or outcome, which is "the innovation" which is not only important but an essential element for any organization seeking to sustain. Methodology of the paper is fit to data, and analysis done by the authors is according to the requirements of paper title.

**Answer: Thank you very much respected Reviewer # 1 for your positive remarks and recommendation of acceptance of our paper.**
